# A mucosal recovery software tool for endoscopic submucosal dissection in early gastric cancer

**DOI:** 10.3389/fmed.2022.1001383

**Published:** 2022-12-07

**Authors:** Yinuo Zhao, Huogen Wang, Yanyan Fan, Chaohui Jin, Qinwei Xu, Jiyong Jing, Tianqiao Zhang, Xuedong Zhang, Wanyuan Chen

**Affiliations:** ^1^Department of Pathology, Rizhao People’s Hospital, Rizhao, China; ^2^Hithink RoyalFlush Information Network Co., Ltd., Hangzhou, China; ^3^College of Computer Science and Technology, Zhejiang University, Hangzhou, China; ^4^Department of Pathology, Liaocheng People’s Hospital, Liaocheng, China; ^5^Endoscopy Center, Shanghai East Hospital, Tongji University School of Medicine, Shanghai, China; ^6^Department of Medical Education & Simulation Center, Zhejiang Provincial People’s Hospital, People’s Hospital of Hangzhou Medical College, Hangzhou, Zhejiang, China; ^7^Hangzhou No.14 High School, Hangzhou, Zhejiang, China; ^8^Cancer Center, Department of Pathology, Zhejiang Provincial People’s Hospital, Affiliated People’s Hospital, Hangzhou Medical College, Hangzhou, Zhejiang, China

**Keywords:** mucosal recovery map, artificial intelligence, endoscopic submucosal dissection, early gastric cancer, Pathology Helper

## Abstract

**Background:**

Due to the limited diagnostic ability, the low detection rate of early gastric cancer (EGC) is a serious health threat. The establishment of the mapping between endoscopic images and pathological images can rapidly improve the diagnostic ability to detect EGC. To expedite the learning process of EGC diagnosis, a mucosal recovery map for the mapping between ESD mucosa specimen and pathological images should be performed in collaboration with endoscopists and pathologists, which is a time-consuming and laborious work.

**Methods:**

20 patients at the Zhejiang Provincial People’s Hospital, Affiliated People’s Hospital of Hangzhou Medical College from March 2020 to July 2020 were enrolled in this study. We proposed the improved U-Net to obtain WSI-level segmentation results, and the WSI-level results can be mapped to the macroscopic image of the specimen. For the convenient use, a software pipeline named as “Pathology Helper” for integration the workflow of the construction of mucosal recovery maps was developed.

**Results:**

The MIoU and Dice of our model can achieve 0.955 ± 0.0936 and 0.961 ± 0.0874 for WSI-level segmentation, respectively. With the help of “Pathology Helper”, we can construct the high-quality mucosal recovery maps to reduce the workload of endoscopists and pathologists.

**Conclusion:**

“Pathology Helper” will accelerate the learning of endoscopists and pathologists, and rapidly improve their abilities to detect EGC. Our work can also improve the detection rate of early gastric cancer, so that more patients with gastric cancer will be treated in a timely manner.

**Core tip:** In this article, we present a new approach to construct the high-quality mucosal recovery maps. We use approximately 20,000 patches to train a deep segmentation network to distinguish cancerous and intestinal metaplasia regions from normal ones. We also develop a mucosal recovery software tool to generates high-quality mucosal recovery maps. In clinical application, this technique can greatly reduce the workload of endoscopists and pathologists and rapidly improve their abilities to detect EGC.

## Introduction

Gastric cancer (gastric carcinoma) is a malignant tumor originating from the gastric mucosal epithelium. In 2018, there were 1,033,701 new cases and 782,685 deaths due to gastric cancer, making it the 6th most commonly diagnosed and 3rd most fatal cancer worldwide (1). In 2015, 42% of the new cases of gastric cancer in the world occurred in China, representing a heavy disease burden of gastric cancer in the country (2). The prognosis of gastric cancer depends largely on the tumor stage. The 5-year survival rate for patients with early gastric cancer is 85–100% with endoscopic submucosal dissection (ESD) operation, while the 5-year survival rate for advanced gastric cancer is <10% (3). However, the early detection rate of gastric cancer is very low. Early detection, diagnosis, and treatment can effectively reduce the mortality of gastric cancer and improve the prognosis after timely treatment. In recent years, with the growth of public health awareness and the popularity of gastroscopy, there was an increase in the number of early gastric cancer detections, but not in the rate of EGC detection. The low rate of diagnosis of EGC may be due to the limited abilities in EGC diagnosis (4).

To expedite the learning process of EGC diagnosis, a mucosal recovery map for the mapping between ESD mucosa specimen and pathological images should be performed in collaboration with endoscopists and pathologists. The mucosal recovery map can show the size, boundary, depth of infiltration, and lymphatic vascular invasion of the lesion. However, it is a time-consuming and laborious work to prepare a mucosal recovery map. To finish a mucosal recovery map, the tumor area should be marked in each slide, and the tumor area should be mapped to the ESD mucosa specimen. If the lesions are large and irregular, it can take many hours to reconstruct a case (5, 6).

Fortunately, the rapid development of deep learning technology provides new ideas to construct mucosal recovery map. Recently, deep learning has been widely used in medical applications, such as computed tomography denoising (7), cell segmentation (8), COVID-19 diagnosis (9), histopathology image classification (10), and breast cancer diagnosis (11). Deep learning can automatically learn task-specific features directly from the data, which can dramatically shorten the time for data processing. In this study, a novel method is proposed for the construction of mucosal recovery maps based on deep learning which can reduce the work intensity of pathologists.

## Materials and methods

**FIGURE 1:**
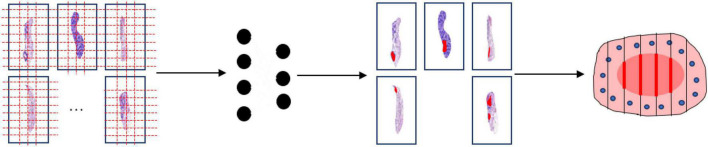
Work flow of the segmentation model. The whole slide images are split into patches **(far left)**. Then the patch-level annotation is obtained with the trained segmentation model **(near left)**. The patch-level annotation is mapped back to the WSI-level annotation based on their original location **(near right)**. Finally, these WSI-level annotations are shown on an image of the entire specimen.

This study was approved by the Ethics Committee of the Zhejiang Provincial People’s Hospital, Affiliated People’s Hospital of Hangzhou Medical College with the informed consent waived. The proposed method for the construction of mucosal recovery maps can be broken down into the following steps: (1) ESD postoperative specimens processing, (2) Pathological Image Segmentation, and (3) Sections mapping. The workflow was shown in Figure 1.

### Endoscopic submucosal dissection postoperative specimens processing

**FIGURE 2:**
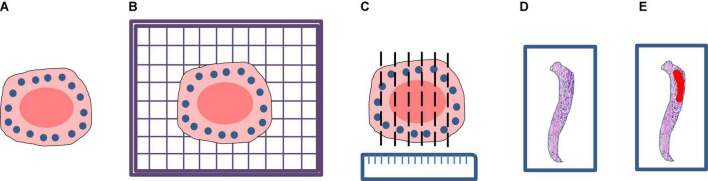
Processing flow of endoscopic submucosal dissection specimen: **(A)** Stretching and fixation; **(B)** macroscopic photography on a cork plate; **(C)** sectioning of the fixed specimen; **(D)** scanning; **(E)** annotation.

A total of 20 patients at the Zhejiang Provincial People’s Hospital, Affiliated People’s Hospital of Hangzhou Medical College from March 2020 to July 2020 were enrolled in this study. All patients were diagnosed with EGC and treated with ESD resection. After ESD resection, all resected specimens were processed according to the guidelines of ESD (12). This procedure included stretching of the fresh specimen, fixation in formalin, sectioning of the fixed specimen, and macroscopic photography before and after sectioning. Firstly, the fresh specimen was stretched and pinned at outer borders upon a cork plate with standard pins, and a macroscopic image of the specimen was taken. Then the specimen was immediately fixed through immersion in 10% formalin for 24∼48 h and a second macroscopic image was taken. Finally, the fixed specimen was cut and sectioned into small sections at intervals of 2.0∼3.0 mm and a third macroscopic image was taken. After the pathological section made, all the sections are scanned into digital WSIs with a Motic scanner. The complete procedure is shown in Figure 2.

### Pathological image segmentation

When a WSI is prepared properly, pathological image segmentation is the most critical step for the construction of mucosal recovery maps. In this study, a novel segmentation network is proposed for pathological image segmentation. The segmentation network can be broken down into the following steps: (1) Data annotation and preprocessing, and (2) Network construction and training.

### Data annotation and preprocessing

**FIGURE 3:**
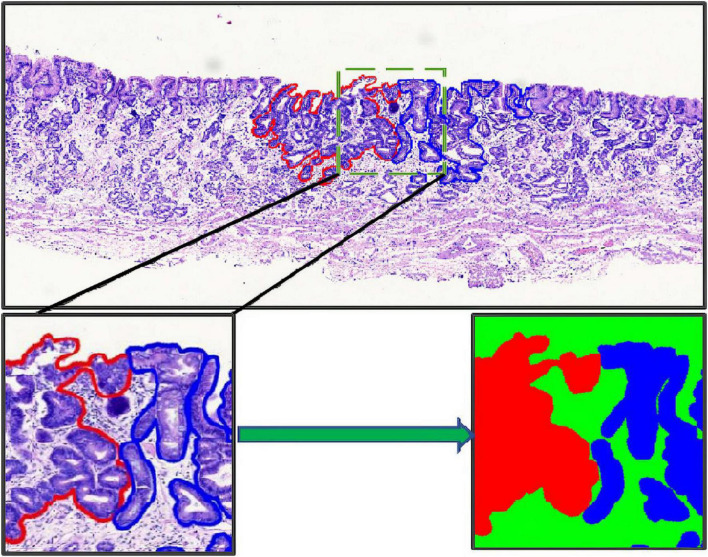
Whole slide image annotation. **(Above and lower left)** Endoscopic submucosal dissection specimen with cancerous regions outlined in red, and intestinal metaplasia regions in blue. **(Lower right)** Corresponding masks for using in deep learning.

**FIGURE 4:**
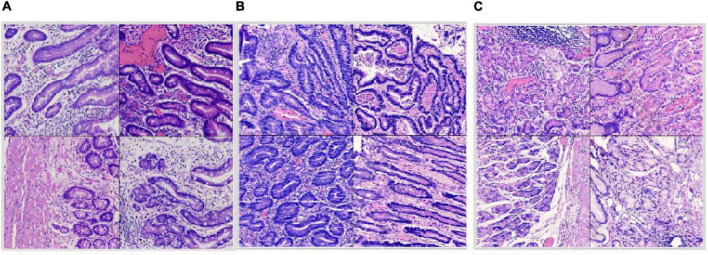
Split dataset: **(A)** Intestinal metaplasia region patches; **(B)** cancerous region patches; **(C)** normal region patches.

The annotation work was carried out according to the Japanese classification of gastric carcinoma: 3rd English edition (13). All WSIs were manually labeled by a group of surgical pathologists by drawing around the cancerous regions (CR) and intestinal metaplasia regions (IR) with red and blue masks, respectively (Figure 3). These masks were modified, confirmed, and verified by another group of pathologists. In the corresponding mask generated, the cancerous regions, intestinal metaplasia regions, and normal mucosa regions (NR) were shown as red, blue, and green, respectively. Then, all annotated WSIs was divided into a training set and a testing set. The training set contained 112 WSIs from 11 patients, and the testing set contained 48 WSIs from 9 patients. Due to the limitations of GPU memory, all WSIs and corresponding masks were split into 512 × 512 pixel patches at 10x magnification (see as Figure 4), and all blank images were removed from the training set. There were 21,799 patches left in the training set and 9,784 patches left in the testing set. The overview of the dataset was shown in Table 1. Random oversampling was adopted for overcome the unbalance between the lesion area and normal area.

**TABLE 1:** The overview of the dataset for network training.

	Training set	Testing set
Cases	11	9
WSIs	112	48
Patches	21,799	9,784

### Network construction and training

**FIGURE 5:**
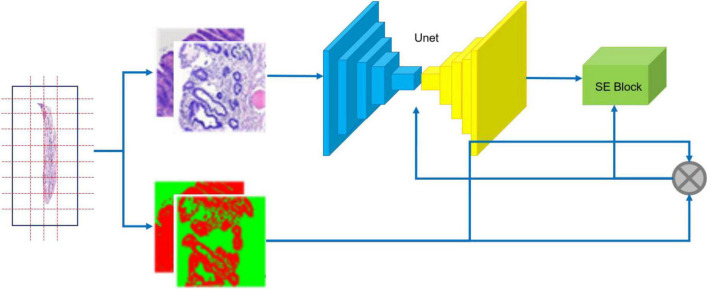
Training flow of CNN. The specimen **(left)** is photographed; the resulting whole slide images are annotated **(middle)**; image-label pairs are fed into the segmentation network **(right)**, which consists of the U-Net and the Squeeze and Excitation (SE) Block.

Our segmentation network incorporates an SE block (14) into U-Net (15) as shown in Figure 5. U-Net is one of the famous Fully Convolutional Networks (FCNs) (16) used in biomedical image segmentation. The image-label pairs in the training set are fed into the segmentation network for training.

**FIGURE 6:**
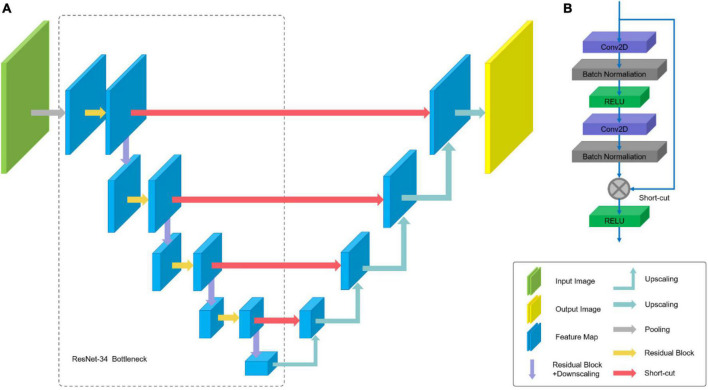
Architecture of U-Net: **(A)** U-Net-like architecture build with pre-trained ResNet-34; **(B)** residual Block.

The ResNet-34 framework is employed as the backbone of U-Net. The architecture of our segmentation network is shown in Figure 6. The special residual blocks (Figure 6B) in ResNet are made up of several convolutional layers with the same number of output channels. Each convolutional layer is followed by a batch normalization layers and a rectified linear unit (ReLU). Then, a shortcut connection and element-wise addition is performed between input and output layers of the block, which make the network easier to optimize (17). Further, a Squeeze-and-Excitation (SE) block is incorporated into U-Net to boost the segmentation performance with increased generalization ability by exploiting adaptive channel-wise feature recalibration (14).

The loss function of our segmentation network is the combination of Jaccard distance loss (18) and cross-entropy loss (8). The loss function can be formulated as follows:


(1) $$l=l_{\mathrm{Jaccard}\,\mathrm{distance}}+l_{\mathrm{cross}\,\mathrm{entropy}}$$

(2) $$l_{\mathrm{Jaccard}\,\mathrm{distance}}=1-\frac{\text{Intersection}}{\text{Union}}=1-\frac{1}{N}\,\sum_{i=1}^{N}\frac{p_{i}\times y_{i}}{p_{i}+y_{i}-p_{i}\times y_{i}}$$

(3) $$l_{\mathrm{cross}\,\mathrm{entropy}}=-\sum_{i}^{N}p_{i}\,\text{log}\,\left(y_{i}\right)$$

In the formula (2) and (3), *y*_*i*_ and *p*_*i*_ are the *i*-th pixels of the labels and predictions, respectively, and *N* is the number of image pixels.

The network was trained using Adam optimizer with learning rate 3 × 10^–4^ for 50 epochs with a batch size of 4. The input size of our network was 512 × 512 pixels. To prevent overfitting, data augmentation was operated on all image-label pairs including rotation (rotation angle range 0∼359°), cropping (vertical and horizontal shift range in 0∼50 pixels), and vertical and horizontal flips. The network is implemented with Keras (TensorFlow backend) and trained on single GTX 1080Ti GPU.

### Sections mapping

**FIGURE 7:**
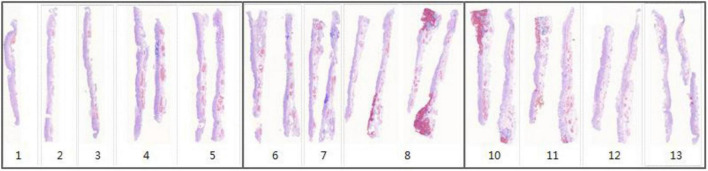
Whole slide image-level thumbnail results of 13 ESD specimens. Blue mark: Intestinal metaplasia. Red mark: Cancerous.

For each WSI in one case, the WSIs are split into 512 × 512 pixel patches. Then the patch-level annotation with the trained segmentation model, and map the patch-level annotation back to the WSI-level annotation based on their original location. Finally, the WSI-level annotation should be mapped back to the ESD specimens (see in Figure 7). Considering that pathological sections may be deformed and atrophied during processing, it was difficult to construct a perfect mucosal recovery map by simple stitching. In this study, GloFlow (19) was employed for slide stitching. GloFlow was a two-stage method for the fusion of pathological image using optical flow-based image registration with global alignment using a computationally tractable graph-pruning approach.

## Results

### Specimen preparation standard

To successfully complete the construction of mucosal recovery maps, the specimen needs to meet the following criteria: (1) The edge of the fixed ESD specimen should not be curly, (2) the surface of specimen should be dry and free of mucus, and (3) the photographs of specimen should be without reflections, and micro-structures should be clearly visible.

### Data and result analysis

We have compared the performance of our model with U-Net on the testing set with 9,784 of patch images. The performance was quantified by using mean intersection over union (MIoU) and Dice Coefficient. MIoU is a standard metric for segmentation purposes, which computes the ratio between the intersection and the union of prediction and ground truth. The Dice Coefficient is two times the Area of Overlap divided by the total number of pixels in both prediction and ground truth.

**FIGURE 8:**
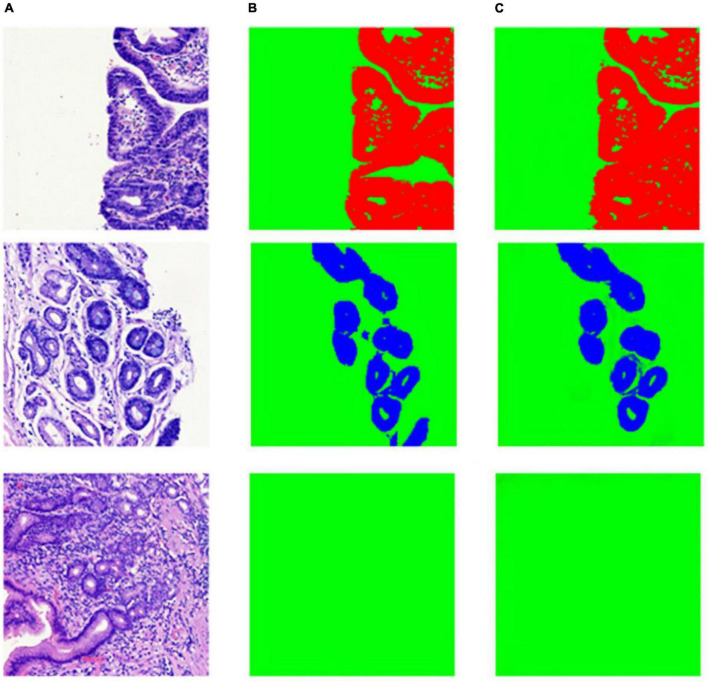
Example of segmentation result in patch-level. **(A)** Whole slide image patches; **(B)** annotation masks; **(C)** deep learning model prediction results.

As shown in Table 2, the segmentation performance of our model and U-Net was listed. From the result in Table 2, our model can achieve better performance than U-Net. This is mostly due to the fact that a Squeeze-and-Excitation (SE) block can boost the segmentation performance with increased generalization ability by exploiting adaptive channel-wise feature recalibration. Some segmentation results and corresponding ground truth are shown in Figure 8.

**TABLE 2:** The segmentation performance of our model and U-Net.

Methods	MIoU	Dice
Our model	0.955 ± 0.0936	0.961 ± 0.0874
U-Net	0.921 ± 0.1761	0.932 ± 0.1585

### The development of “Pathology Helper”

**FIGURE 9:**
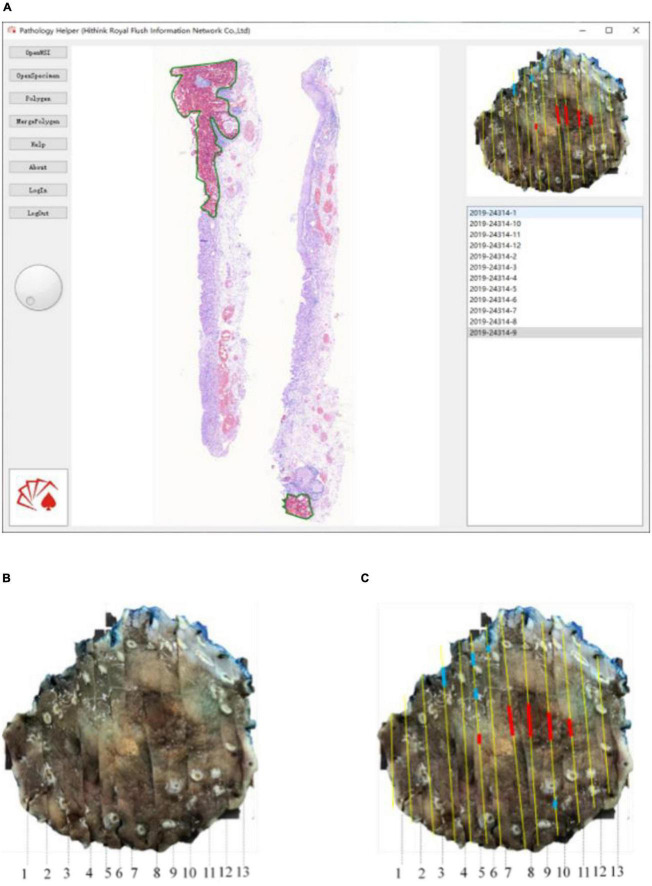
Whole slide image-level results mapping flow: **(A)** Mapping with Pathology Helper software; **(B)** specimen photo; **(C)** mapping result.

For the convenient use, a software pipeline named as “Pathology Helper” for integration the workflow of the construction of mucosal recovery maps was developed. The interface of “Pathology Helper” is shown as Figure 9.

## Conclusion

In the clinical diagnosis and treatment of early gastric cancer, the detection rate (i.e., the number of early gastric cancers as a percentage of the total number of diagnosed gastric cancers) is an important index measuring the level of an endoscopic center. The detection rate varies from place to place in China: It can reach 40% in developed cities along the southeast coast, but is less than 10% in remote areas. The overall detection rate in China is about 15%. Therefore, it is very important to improve the detection rate of early gastric cancer.

Mucosal recovery maps can help pathologists and endoscopists improve their understanding of endoscopy and pathomorphology. However, given a specimen of 6 cm × 5 cm × 0.2 cm and lesion area about 3 cm × 2 cm, it will take about 60 min for a skilled subspecialist in pathology to complete a finely made mucosal recovery map. If the histological classification of the cancer is complex, it may take even longer to complete the task. As a result, many endoscopists are unable to obtain high-quality mucosal recovery maps. In recent years, deep learning has been widely applied in the field of pathological diagnosis, thanks to the popularization of pathological section digitization. In 2017, Esteva et al. (20) used a convoluted neural network to analyze 129,450 pathological images of skin lesions and trained the model to distinguish skin squamous cell carcinoma from seborrheic keratosis, malignant melanoma, and benign nevus with the same accuracy as doctors. In 2019, Kather et al. (21) used a deep residual learning algorithm to identify microsatellite instability (MSI) directly from pathological slices. The accuracy of MSI recognition of colorectal cancer was 84%. There have also been artificial intelligence-assisted diagnostic studies on histopathology, including glioma grade (20), lymphoma classification (21), colorectal cancer polyp classification (22), and prostate cancer diagnosis (23). All these works matched or even went beyond the diagnostic level attained by human pathologists.

In this study, we design a novel segmentation network for pathological image segmentation. Starting with WSIs labeled by surgical pathologists in early gastrointestinal cancer, we trained a novel segmentation network for the automatical annotation of WSIs. Our segmentation network incorporates an SE block (14) into U-Net (15), one of the famous Fully Convolutional Networks (FCNs) (16) used in biomedical image segmentation. U-Net has had many successful applications, such as brain image segmentation (24), liver image segmentation (25), and cell counting, detection, and morphometry (26). However, it fails to take the differentiate between channel-wise features. In general, the SE block was proposed to be placed in InceptionNet (27) and ResNet (17) for boosting performance in classification and object detection *via* feature recalibration. Accordingly, we incorporate it into U-Net to boost the segmentation performance with increased generalization ability by exploiting adaptive channel-wise feature recalibration. The experiments show that our proposed network has better performance than U-Net alone. After pathological image segmentation, the WSI-level segmentation result is mapped back to the ESD specimen with the help of a mucosal recovery software tool “Pathology Helper”.

“Pathology Helper” can help in the production of high-quality mucosal recovery maps. This will accelerate the learning of endoscopists and pathologists, and rapidly improve their abilities to detect EGC. Our work can also improve the detection rate of early gastric cancer, so that more patients with gastric cancer will be treated in a timely manner. However, this software tool still had several limitations. For example, the pathological image segmentation network was developed and trained on the dataset from a single large academic institution, which lacked multi-center or external data validation. Future research is required to determine if the same model trained can achieve high performance on larger or multi-institutional datasets.

## Data availability statement

The raw data supporting the conclusions of this article will be made available by the authors, without undue reservation.

## Ethics statement

The studies involving human participants were reviewed and approved by the Ethics Committee of the Zhejiang Provincial People’s Hospital, Affiliated People’s Hospital of Hangzhou Medical College. The ethics committee waived the requirement of written informed consent for participation.

## Author contributions

WC, XZ, and YZ: study conception and design. WC, XZ, YZ, YF, QX, and JJ: acquisition of data. HW, CJ, JJ, and TZ: analysis and interpretation of data and statistical analysis. YZ and HW: drafting of the manuscript. WC and XZ: critical revision of the manuscript for important intellectual content, and administrative, technical, or material support. WC: supervision. All authors were involved in writing the manuscript and had final approval of the submitted and published versions.

## References

[1] Bray F Ferlay J Soerjomataram I Siegel RL Torre LA Jemal A. Global cancer statistics 2018: GLOBOCAN estimates of incidence and mortality worldwide for 36 cancers in 185 countries. *CA Cancer J Clin.* (2018) 68:394–424. 10.3322/caac.21492 30207593

[2] Chen W Zheng R Baade PD Zhang S Zeng H Bray F Cancer statistics in China, 2015. *CA Cancer J Clin.* (2016) 66:115–32. 10.3322/caac.21338 26808342

[3] Orditura M Galizia G Sforza V Gambardella V Fabozzi A Laterza MM Treatment of gastric cancer. *World J Gastroenterol.*(2014) 20:1635. 10.3748/wjg.v20.i7.1635 PMC3930964 24587643

[4] Zhang Q Chen ZY Chen CD Liu T Tang XW Ren YT Training in early gastric cancer diagnosis improves the detection rate of early gastric cancer: an observational study in China. *Medicine.* (2015) 94:e384. 10.1097/MD.0000000000000384 25590840 PMC4602560

[5] Reggiani Bonetti L Manta R Manno M Conigliaro R Missale G Bassotti G Optimal processing of ESD specimens to avoid pathological artifacts. *Tech Coloproctol.* (2018) 22:857–66. 10.1007/s10151-018-1887-x 30560321

[6] Ebigbo A Probst A Messmann H Märkl B Nam-Apostolopoulos YC. Topographic mapping of a specimen after endoscopic submucosal dissection. *Endoscopy Int Open.* (2019) 7:E521–4. 10.1055/a-0846-2043 31041368 PMC6447407

[7] Gholizadeh-Ansari M Alirezaie J Babyn P. Deep learning for low-dose CT denoising using perceptual loss and edge detection layer. *J Digit Imaging.* (2020) 33:504–15. 10.1007/s10278-019-00274-4 31515756 PMC7165209

[8] Akram SU Kannala J Eklund L Heikkilä J. Cell segmentation proposal network for microscopy image anlysis. In: *Proceedings of the Medical Image Computing and Computer-Assisted Intervention*. Athens: Springer (2016). p. 21–9.

[9] Rahaman MM Li C Yao Y Kulwa F Rahman MA Wang Q Identification of COVID-19 samples from chest X-Ray images using deep learning: a comparison of transfer learning approaches. *J X-ray Sci Technol.* (2020) 28:821–39. 10.3233/XST-200715 32773400 PMC7592691

[10] Haoyuan C Chen L Xiaoyan L Ge W Weiming H Yixin L GasHis-Transformer: a multi-scale visual transformer approach for gastric histopathological image detection. *Pattern Recognit.* (2022) 130:108827. 10.1016/j.patcog.2022.108827

[11] Wang D Khosla A Gargeya R Irshad H Beck AH. Deep learning for identifying metastatic breast cancer. *arXiv.* (Preprint). (2016). 10.48550/arXiv.1606.05718 35895330

[12] Ono H Yao K Fujishiro M Oda I Uedo N Nimura S Guidelines for endoscopic submucosal dissection and endoscopic mucosal1 resection for early gastric cancer. *Digest Endoscopy.* (2021) 33:4–20. 10.1111/den.13883 33107115

[13] Japanese Gastric Cancer Association [JGCA]. Japanese classification of gastric carcinoma: 3rd english edition. *Gastric Cancer.* (2011) 14:101–12. 10.1007/s10120-011-0041-5 21573743

[14] Hu J Shen L Albanie S Sun G Wu E. Squeeze-and-excitation networks. *IEEE Trans Pattern Anal Mach Intell.* (2020) 42:2011–23. 10.1109/TPAMI.2019.2913372 31034408

[15] Ronneberger O Fischer P Brox T. U-net: Convolutional networks for biomedical image segmentation. *International conference on medical image computing and computer-assisted intervention.* Cham: Springer (2015). p. 234–41.

[16] Shelhamer E Long J Darrell T. Fully convolutional networks for semantic segmentation. *IEEE Trans Pattern Anal Mach Intell.* (2017) 39:640–51. 10.1109/TPAMI.2016.2572683 27244717

[17] He K Zhang X Ren S Sun J. Deep residual learning for image recognition. *Proceedings of the IEEE conference on computer vision and pattern recognition.* Las Vegas, NV: IEEE (2016). p. 770–8.

[18] Kosub S. A note on the triangle inequality for the jaccard distance. *Pattern Recognit Lett.* (2019) 120:36–8.

[19] Krishna V Joshi A Bulterys PL Yang E Ng AY Rajpurkar P. GloFlow: global image alignment for creation of whole slide images for pathology from video. *arXiv.* (Preprint). (2020). arXiv:2010.15269.

[20] Esteva A Kuprel B Novoa RA Ko J Swetter SM Blau HM Dermatologist-level classification of skin cancer with deep neural networks. *Nature.* (2017) 542:115–8. 10.1038/nature21056 28117445 PMC8382232

[21] Kather JN Pearson AT Halama N Jäger D Krause J Loosen SH Deep learning can predict microsatellite instability directly from histology in gastrointestinal cancer. *Nat Med.* (2019) 25:1054–6. 10.1038/s41591-019-0462-y 31160815 PMC7423299

[22] Ertosun MG Rubin DL. Automated grading of gliomas using deep learning in digital pathology images: a modular approach with ensemble of convolutional neural networks. *AMIA Annu Symp Proc.* (2015) 2015:1899–908. 26958289 PMC4765616

[23] Janowczyk A Madabhushi A. Deep learning for digital pathology image analysis: a comprehensive tutorial with selected use cases. *J Pathol Informat.* (2016) 7:29. 10.4103/2153-3539.186902 27563488 PMC4977982

[24] Kong X Sun G Wu Q Liu J Lin F. Hybrid pyramid u-net model for brain tumor segmentation. *International conference on intelligent information processing.* Cham: Springer (2018). p. 346–55.

[25] Liu Z Song YQ Sheng VS Wang L Jiang R Zhang X Liver CT sequence segmentation based with improved U-Net and graph cut. *Expert Syst Appl.* (2019) 126:54–63.

[26] Falk T Mai D Bensch R Çiçek Ö Abdulkadir A Marrakchi Y U-Net: deep learning for cell counting, detection, and morphometry. *Nat Methods.* (2019) 16:67–70. 30559429 10.1038/s41592-018-0261-2

[27] Szegedy C Vanhoucke V Ioffe S Shlens J Wojna Z. Rethinking the inception architecture for computer vision. *Proceedings of the IEEE conference on computer vision and pattern recognition.* Las Vegas, NV: IEEE (2016). p. 2818–26.

